# Case Report: Successful treatment of pediatric alopecia universalis with ritlecitinib after failure of baricitinib

**DOI:** 10.3389/fmed.2025.1675062

**Published:** 2025-12-18

**Authors:** Xiaoqing Lv, Ying Chen, Shi-Fan Ruan, Qiuyun Xu, Jing Mao, Li Zhu, Wenxuan Xia, Siyi Bao, Zheyu Zhuang, Lihong Chen, Bo Cheng, Chao Ji

**Affiliations:** 1 Department of Dermatology, The First Affiliated Hospital of Fujian Medical University, Fuzhou, Fujian, China; 2 Key Laboratory of Skin Cancer of Fujian Higher Education Institutions, The Fujian Medical University, Fuzhou, Fujian, China; 3 Fujian Dermatology and Venereology Research Institute, The First Affiliated Hospital, Fujian Medical University, Fuzhou, Fujian, China; 4 Fujian Provincial Clinical Research Center for Immune Skin Diseases, The First Affiliated Hospital of Fujian Medical University, Fuzhou, Fujian, China; 5 Department of Dermatology, Beijing Anzhen Nanchong Hospital, Capital Medical University & Nanchong Central Hospital, Nanchong, Sichuan, China; 6 School of Medicine, Fuzhou University, Fuzhou, Fujian, China

**Keywords:** alopecia areata, alopecia universalis, janus kinase inhibitors, ritlecitinib, treatment

## Abstract

Alopecia areata is characterized by difficult treatment and easy recurrence, especially posing significant physical and psychological distress to children. However, current treatment options for Pediatric alopecia areata are relatively limited, with generally poor efficacy and numerous side effects. We report the first case of a Pediatric patient transitioning from baricitinib therapy to ritlecitinib, resulting in favorable clinical outcomes. We believe that the result represent a meaningful advance in our understanding of how to treat children with chronic severe AA and feel that this would be of substantial interest to the broad readership of Frontiers in medicine.

## Introduction

Alopecia areata (AA) is a recurrent autoimmune disorder characterized by non-scarring hair loss, ranging from localized patches to alopecia totalis (AT) or alopecia universalis (AU), and is often accompanied by significant psychological burden, especially in children (1, 2). Current treatments, such as corticosteroids and immunosuppressants, have limited efficacy and potential side effects in the pediatric population (3). Here, we describe a child with AA who was initially limited response to baricitinib while significantly benefited from switching to ritlecitinib, indicating ritlecitinib may be a promising treatment option in refractory pediatric cases.

## Case report

**Figure 1:**
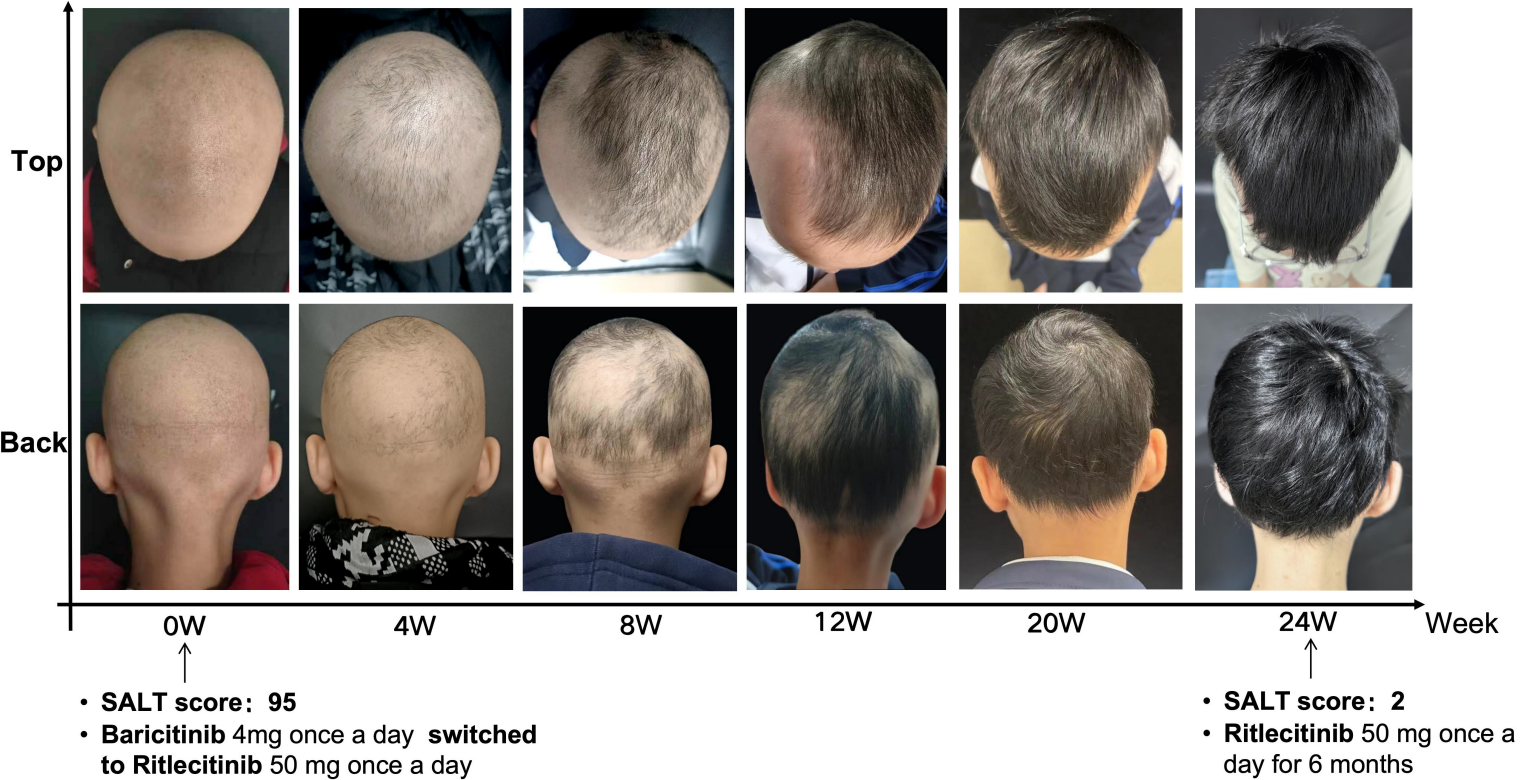
Improvement of AU after switching to ritlectinib.

A 10-year-old girl presented to our dermatology clinic with a 4-year history of AU and complained of mild nail deformity and significant psychosocial distress, manifesting as anxiety symptoms and social avoidance reported by her caregivers. Her medical history was otherwise unremarkable. Previous treatments, including topical and systemic corticosteroids as well as oral baricitinib, had elicited poor responses. Specifically, treatment with baricitinib 4 mg daily resulted in the regrowth of her eyebrows and eyelashes but failed to stimulate scalp hair growth after 9 months. Given the limited response and the increasing psychological burden on the patient, her caregivers requested consideration of alternative therapeutic options. Subsequently, the patient was prescribed 50 mg of ritlecitinib daily. Following a 6-month treatment with ritlecitinib, the patient exhibited a notable decrease in both Severity of Alopecia Tool (SALT) score, from 95 to 2. Based on the Children's Dermatology Life Quality Index (CDLQI), the patient's score improved from 30 to 5 after treatment, showing significant improvement. Notably, in the “Leisure” and “Personal Relationships” domains, she overcame avoidance of activities and social exclusion, re-engaging in daily activities and peer interactions. These results demonstrate that treatment not only alleviated symptoms but also enhanced her psychosocial functioning and quality of life. These improvements suggest significant enhancement in the patient's alopecia-related hair loss and quality of life, attributable to ritlecitinib therapy. Remarkably, post-treatment observations revealed substantial regrowth of dark hair in the temporal and occipital regions, the emergence of vellus hairs in other regions, and an improvement in nail deformities (Figure 1). We conducted a total follow-up period of 26 months, including 18 months of daily ritlecitinib treatment. After complete and stable scalp hair regrowth was achieved, the medication was continued for an additional 3 months at an alternate-day dosing regimen to consolidate remission. The drug was subsequently discontinued, and the patient remained in remission, with no evidence of relapse during the 5-month post-treatment follow-up. Mild acne was observed during treatment, while no other adverse reactions were noted, suggesting a sustained clinical benefit and good tolerability of the medication. Safety was rigorously monitored throughout the treatment course. Regular laboratory assessments, including complete blood count, blood biochemistry, coagulation function, and IgE, revealed no abnormalities throughout the treatment period.

## Discussion

AA is a cell-mediated autoimmune disorder involving cytotoxic T-cell response that targets hair follicles. Recent studies have elucidated the role of the JAK/STAT signaling pathway in promoting the inflammatory response around hair follicles (4). The JAK family comprises four members: JAK1, JAK2, JAK3, and TYK2 (5). The various pharmacological inhibition of JAK subtypes may enhance the suppression of AA-relevant cytokines, such as IL-15 and IFN-γ, potentially accounting for variations in clinical efficacy observed. The FDA has approved two medications for AA, both demonstrating safety and effectiveness: the JAK1/2 inhibitor baricitinib (over 18 years old) and the JAK3 inhibitor ritlecitinib (over 12 years old) for treat AA.

In our case, the patient initially experienced unsatisfactory outcomes from baricitinib. During a literature search, we found that ritlecitinib has proven to be an effective treatment with a favorable benefit–risk ratio in adolescents and adults. In a phase 3 randomized controlled trial, 25%−50% of adolescents with severe AA treated with ritlecitinib achieved a SALT score ≤ 20 at Week 48, significantly outperforming placebo (6). In a recent retrospective analysis of 18 pediatric patients with alopecia areata, Wang et al. reported that ritlecitinib demonstrated favorable short-term efficacy and tolerability in children under 12 years of age with severe disease (7). Importantly, regarding the management of refractory cases, emerging evidence supports the strategy of JAK inhibitor conversion therapy. Chen et al. specifically evaluated the efficacy of switching from baricitinib to ritlecitinib in patients with severe AA who had an inadequate response to baricitinib. They reported that 60% of patients achieved significant regrowth (SALT50) after the switch, providing strong evidence that this specific sequential strategy is effective (8). Our case extends these findings into the pre-adolescent population. Her successful outcome—achieving near-complete regrowth after switching—validates this mechanism-based conversion strategy in younger children. These findings suggest that the distinct kinase selectivity profiles (e.g., JAK1/2 vs. JAK3/TEC) may offer different therapeutic pathways, making ritlecitinib a valuable rescue option for pediatric patients who do not respond to first-line JAK inhibitors. Treatment was generally well tolerated, with most adverse events being mild to moderate (e.g., headache, acne, nasopharyngitis), and no signal for serious toxicity or growth impairment. Accordingly, in view of the patient's inadequate response to baricitinib, the supportive efficacy and safety profile of ritlecitinib, its more suitable age indication, and existing evidence suggesting potential benefit from JAK-to-JAK switching, initiation of ritlecitinib was considered clinically justified. As supported by clinical data, ritlecitinib responses appear time-dependent, with meaningful improvement typically observed within the first few months and progressive benefit reported with continued therapy for 24–48 weeks (4). Long-term extension studies further support ongoing treatment to sustain disease control.

The successful switch in treatment may be attributed to the specific action of JAK3-selective inhibitors. These inhibitors specifically target γc chain receptor signaling while sparing the signaling pathways of immunoregulatory cytokines, such as IL-10R (TYK2/JAK1), IL-27R (JAK1/JAK2), and IL-35R (JAK1/JAK2) (9), which may contribute to preventing autoimmunity in the hair follicle. This potential specificity makes selective JAK3 inhibitors a more potent strategy compared to pan-JAK inhibitors. Additionally, advantages of using ritlecitinib may avoid side effects of pan-JAK inhibitors, such as increased cholesterol and liver enzymes, and those related to JAK2 inhibition, including thrombocytopenia and anemia. In summary, JAK3 inhibitors could offer better efficacy/safety ratios.

In this case, ritlecitinib treatment resulted in significant hair regrowth, which subsequently led to a marked improvement in the patient's psychosocial well-being. Based on this favorable clinical outcome and safety profile, ritlecitinib appears to be an effective and well-tolerated option for children with chronic moderate-to-severe AA, offering a promising alternative for those resistant to baricitinib. However, the limitations of this case include a relatively short follow-up period. Finally, we acknowledge that the 9-month duration of initial baricitinib therapy may not have been sufficient to capture potential late responders.

We acknowledge the legal guardian of the patient for providing informed consent for the publication of the photographs.

## Data Availability

The original contributions presented in the study are included in the article/Supplementary material, further inquiries can be directed to the corresponding author/s.
