# Supplementary material for: Case Report: Successful treatment of pediatric alopecia universalis with ritlecitinib after failure of baricitinib
Source: Front Med (Lausanne). 2025 Dec 18;12:1675062. doi: 10.3389/fmed.2025.1675062 (PMC12756114; doi:10.3389/fmed.2025.1675062)
Supplement: Supplementary file 1 [file Table_1.docx]

**Supplementary Table S1.** Longitudinal assessment of clinical efficacy based on Severity of Alopecia Tool (SALT) scores and regional scalp breakdown during ritlecitinib treatment.

| **SALT Score**  **Time(week)** | **W 0** | **W 4** | **W 8** | **W 12** | **W 20** | **W 24** |
| --- | --- | --- | --- | --- | --- | --- |
| Vertex (Max 40) | 38 | 34 | 24 | 12 | 3 | 1 |
| Posterior (Max 24) | 23 | 20 | 14 | 6 | 3 | 1 |
| Right profile (Max 18) | 17 | 15 | 11 | 6 | 2 | 0 |
| Left profile (Max 18) | 17 | 15 | 11 | 6 | 2 | 0 |
| **Total Score (0–100)** | **95** | **84** | **60** | **30** | **10** | **2** |

Note: The SALT score is calculated as the sum of the percentage of hair loss in each of the four scalp areas multiplied by the relative surface area of that region. Values presented represent the weighted score contribution of each region to the total score. Lower scores indicate greater hair regrowth.
