# Supplementary material for: Case Report: Successful treatment of pediatric alopecia universalis with ritlecitinib after failure of baricitinib
Source: Front Med (Lausanne). 2025 Dec 18;12:1675062. doi: 10.3389/fmed.2025.1675062 (PMC12756114; doi:10.3389/fmed.2025.1675062)
Supplement: Supplementary file 2 [file Table_2.docx]

**Supplementary Table S2**. Longitudinal evaluation of psychosocial well-being using Children’s Dermatology Life Quality Index (CDLQI) total scores and itemized domain analysis.

| **Assessment Parameter**  **Time (Week)** | **W 0** | **W 4** | **W 8** | **W12** | **W 20** | **W24** |
| --- | --- | --- | --- | --- | --- | --- |
| 1. Itchy/sore/painful skin | 3 | 2 | 1 | 1 | 1 | 1 |
| 2. Embarrassment/self-consciousness | 3 | 3 | 2 | 1 | 1 | 1 |
| 3. Friendships | 3 | 3 | 2 | 1 | 0 | 0 |
| 4. Clothes/shoes | 3 | 3 | 2 | 1 | 1 | 0 |
| 5. Social/Leisure | 3 | 3 | 2 | 1 | 0 | 0 |
| 6. Sport | 3 | 2 | 2 | 1 | 0 | 0 |
| 7. School/Holiday | 3 | 3 | 2 | 1 | 0 | 0 |
| 8. Nicknames/Bullying | 3 | 2 | 2 | 1 | 0 | 0 |
| 9. Sleep | 3 | 3 | 2 | 1 | 1 | 1 |
| 10. Treatment problem | 3 | 3 | 2 | 2 | 2 | 2 |
| **Total Score (0–30)** | **30** | **27** | **19** | **11** | **6** | **5** |

Note: CDLQI scores for each question range from 0 to 3 (0 = not at all, 1 = only a little, 2 = quite a lot, 3 = very much). The total score ranges from 0 to 30, with higher scores indicating a greater impairment of quality of life. The significant reduction in scores for questions 3–7 (social and school domains) from Week 12 onwards correlates with the period of substantial hair regrowth observed in Table 1.
